# Predicting MCI to AD Conversation Using Integrated sMRI and rs-fMRI: Machine Learning and Graph Theory Approach

**DOI:** 10.3389/fnagi.2021.688926

**Published:** 2021-07-30

**Authors:** Tingting Zhang, Qian Liao, Danmei Zhang, Chao Zhang, Jing Yan, Ronald Ngetich, Junjun Zhang, Zhenlan Jin, Ling Li

**Affiliations:** Key Laboratory for NeuroInformation of Ministry of Education, High-Field Magnetic Resonance Brain Imaging Key Laboratory of Sichuan Province, Center for Information in Medicine, School of Life Sciences and Technology, University of Electronic Science and Technology of China, Chengdu, China

**Keywords:** resting-state fMRI, structural MRI, mild cognitive impairment, graph theoretical analysis, machine learning, classification

## Abstract

**Background:**

Graph theory and machine learning have been shown to be effective ways of classifying different stages of Alzheimer’s disease (AD). Most previous studies have only focused on inter-subject classification with single-mode neuroimaging data. However, whether this classification can truly reflect the changes in the structure and function of the brain region in disease progression remains unverified. In the current study, we aimed to evaluate the classification framework, which combines structural Magnetic Resonance Imaging (sMRI) and resting-state functional Magnetic Resonance Imaging (rs-fMRI) metrics, to distinguish mild cognitive impairment non-converters (MCInc)/AD from MCI converters (MCIc) by using graph theory and machine learning.

**Methods:**

With the intra-subject (MCInc vs. MCIc) and inter-subject (MCIc vs. AD) design, we employed cortical thickness features, structural brain network features, and sub-frequency (full-band, slow-4, slow-5) functional brain network features for classification. Three feature selection methods [random subset feature selection algorithm (RSFS), minimal redundancy maximal relevance (mRMR), and sparse linear regression feature selection algorithm based on stationary selection (SS-LR)] were used respectively to select discriminative features in the iterative combinations of MRI and network measures. Then support vector machine (SVM) classifier with nested cross-validation was employed for classification. We also compared the performance of multiple classifiers (Random Forest, K-nearest neighbor, Adaboost, SVM) and verified the reliability of our results by upsampling.

**Results:**

We found that in the classifications of MCIc vs. MCInc, and MCIc vs. AD, the proposed RSFS algorithm achieved the best accuracies (84.71, 89.80%) than the other algorithms. And the high-sensitivity brain regions found with the two classification groups were inconsistent. Specifically, in MCIc vs. MCInc, the high-sensitivity brain regions associated with both structural and functional features included frontal, temporal, caudate, entorhinal, parahippocampal, and calcarine fissure and surrounding cortex. While in MCIc vs. AD, the high-sensitivity brain regions associated only with functional features included frontal, temporal, thalamus, olfactory, and angular.

**Conclusions:**

These results suggest that our proposed method could effectively predict the conversion of MCI to AD, and the inconsistency of specific brain regions provides a novel insight for clinical AD diagnosis.

## Introduction

Mild cognitive impairment (MCI) is considered a transitional state between normal aging and early Alzheimer’s disease (AD) (Lee et al., 2012). Studies have shown that individuals with MCI tend to develop AD at a rate of about 10–15% per year (Allison et al., 2014), but the probability of a healthy elderly to be diagnosed with AD is only 1∼2% (Bischkopf et al., 2002). If MCI is diagnosed at an early stage, through rehabilitation exercise and medication, the incidence of AD can be reduced by nearly one-third (Golob et al., 2007). Thus, early detection of MCI individuals makes it possible to potentially delay or prevent the transition from MCI to AD. The following are MCI clinical conversion criteria: MCI patients can be divided into MCIc and MCInc, depending on whether they become converted into AD patients within a certain period (for instance, the conversion time could be 36 months, 48 months, etc.). Interestingly, the two types of patients have similar clinical manifestations in the early stage, and the morphological differences of their brain lesions are small. To intervene in the diagnosis and treatment of AD disease earlier, the diagnosis and prediction of MCI disease have been studied from multiple perspectives such as genetics, pathology, and medical imaging. Currently, there are different opinions on biomarkers that can accurately reflect the timeliness of preclinical disease progression. However, no research has established the versatility of such markers using prediction/validation study designs. Furthermore, there are defects and difficulties in the diagnosis and classification of MCI disease development. Therefore, finding high discriminative features and establishing a robust classification mechanism is of clinical significance for the diagnosis and timely treatment of MCI diseases, especially the provision of early warning signs for high-risk MCI patients. This may guide the patients to make rational treatment decisions, and thus, even prevent them from developing AD.

Neuroimaging studies of AD patients have found atrophy of structural tissues, and abnormal connections between brain regions in structure and function (Liu et al., 2012; Dai et al., 2019; Zhang et al., 2019). Especially, neuroanatomical abnormalities have been found to spread from one brain area to another based on distinctive network patterns in neurodegenerative diseases (Yates, 2012; Pandya et al., 2017; Cauda et al., 2018). Eskildsen and his colleagues (Eskildsen et al., 2013) classified MCI and AD using cortical thickness features from structural MRI and achieved accuracies ranging from 70 to 76% depending on the conversion time. Taking advantage of the difference in the time dimension of disease, Li and his colleagues (Li et al., 2012) proposed a 4-D disease classification algorithm based on the thickness of the cerebral cortex. The classification of MCIc and MCInc achieved the highest classification accuracy (81.7%). Since most studies have reported abnormal and inconsistent brain connections, many recent studies have used the construction of a classification framework combining brain networks and machine learning to classify MCI\AD. Raamana and colleagues (Raamana et al., 2015) constructed a brain network based on the difference in cortical thickness, by taking the average clustering coefficient, boundary number, and node degree as features, and using a multi-core Bayes classifier to classify MCIc and MCInc with a classification accuracy of 64%. Our previous study (Wei et al., 2016) proposed a classification framework to distinguish MCIc from MCInc by using MRI and network features and attained the best accuracies of 76.39%.

To improve the classification effect, many studies have been dedicated to fusing different types of data, such as MRI, fMRI, positron emission tomography (PET), cerebrospinal fluid (CSF), and cognitive scoring scales. Liu et al. (2014) proposed a new multi-modal classification method combining PET and MRI with an accuracy of 67.83% for the classification of MCInc and MCIc. While Wee et al. (2012b) used multi-core SVM to integrate diffusion tensor image (DTI) and rs-fMRI functional network features to classify MCI and normal elderly people and obtained a higher classification accuracy of 96.3%, which was 7.4% higher than that of single-mode data. Besides, appropriate feature selection (Zuo et al., 2010; Chu et al., 2012) and frequency division (Wee et al., 2012a; Mascali et al., 2015) have also been proven to effectively improve classification accuracy. One of our recent studies (Zhang et al., 2019) supports this view. Essentially, our earlier study distinguished individuals with EMCI and LMCI using a functional brain network of three frequency bands and three feature selection algorithms, during the Resting States, and obtained 83.87% accuracy using the mRMR algorithm in a slow-5 band. Although most previous studies have investigated the utility of the structural MRI or rs-fMRI for classification of MCIc from MCInc, few studies have used cortical and subcortical measurements extracted from DTI/MRI, and graph measures extracted from rs-fMRI, to classify MCIc and MCInc (Mascali et al., 2015; Hojjati et al., 2018). Besides, previous studies only focused on the classification of the different groups of patients, but whether this kind of classification can truly reflect the changes in the structure and function of the brain regions in disease progression remains unverified.

To address these issues, this study aims to: (i) incorporate multiple structural and functional metrics into a combined graph theoretical and machine learning analysis, to evaluate the efficacy of a classification framework to distinguish MCInc/AD from MCIc. (ii) predict the highly sensitive brain regions of AD conversion, by comparing the difference of the brain regions between MCIc and MCInc, with that between MCIc and AD. Firstly, we proposed structural features including MRI features by FreeSurfer and nodal parameters from thickness network, and functional features derived from constructed functional brain network among time series of the brain regions with three frequency bands (full-band, slow-4, slow-5) at Resting State. Subsequently, we established a weighted network by using a kernel function, and then thresholded it to a binary network at a high discriminative range of sparsity from 8 to 44%. In the current study, the SS-LR and mRMR feature selection algorithms build upon our previous work (Wei et al., 2016; Zhang et al., 2019). We employed novel feature selection algorithms (RSFS) to find effective features, and then trained and tested the SVM classifier for classification. We also tested the reliability and stability of the best classification results by applying multiple classifiers (Random Forest, K-nearest neighbor (KNN), AdaBoost, SVM) by upsampling. Finally, we compared the selected top 10 features from the classification of MCInc vs. MCIc and those from the MCIc vs. AD group. Meanwhile, we also investigated the contribution of each modal to the multi-modal classification to explore the conversion of MCI. We hypothesized that the proposed method will improve the accuracy and the sensitivity of identifying prodromal AD, and that the high-sensitivity brain regions of the two classification groups may be inconsistent. To the best of our knowledge, this is the first study that has used cortical thickness, structural brain network, and sub-frequency functional brain network for this classification (MCInc vs. MCIc, MCIc vs. AD). Besides, another innovation of this study is the employment of the intra-subject and inter-subject design to classify the two groups of patients.

## Materials and Methods

### Participants

Data used in this study were obtained from the Alzheimer’s Disease Neuroimaging Initiative (ADNI) database.^1^ The ADNI was launched in 2003 as a public-private partnership, led by Principal Investigator Michael W. Weiner, MD. The primary goal of ADNI was to test whether serial magnetic resonance imaging (MRI), positron emission tomography (PET), some biological markers, and clinical and neuropsychological assessment can be combined to measure the progression of mild cognitive impairment (MCI) and early Alzheimer’s disease (AD). The demographic data of the datasets are listed in Table 1. A total of 108 participants with full structural and resting-state functional data were collected, but 4 of them failed to pass the data quality control. In the ADNI project, the diagnostic criteria of MCI were as follows: (1) Mini-Mental State Examination (MMSE) scores between 24 and 30. (2) Clinical Dementia Rating (CDR) is 0.5. (3) Memory complaint, objective memory loss measured by education adjusted scores on the Wechsler Memory Scale Logical Memory II. (4) No observable impairment in other cognitive fields, and able to remember the activities of daily life (no dementia).

**TABLE 1:** Characteristics of the participants.

Variable	MCInc (*n* = 55)	MCIc (*n* = 30)	AD (*n* = 19)	*p*-value MCInc vs. MCIc	*p*-value MCIc vs. AD
Gender (M/F)	25/30	16/14	10/9	0.487	0.962
Age	72.01 ± 8.21	74.40 ± 7.19	75.08 ± 6.33	0.186	0.734
MMSE	28.16 ± 1.78^*a*^	27.00 ± 1.88	25.00 ± 2.79^*a*^	0.006	0.004
CDR	0.47 ± 0.12	0.55 ± 0.20	0.84 ± 0.24^*a*^	0.060	<0.001
Education	15.85 ± 2.71	15.80 ± 2.59	16.37 ± 2.36	0.928	0.443

*MMSE, Mini Mental State Examination scores; CDR, Clinical Dementia Rating. A Chi-square test was used for gender comparison. A two-tailed student’s t-test was used to compare age, neuropsychological tests, and education level. ^a^Indicates significance compared to the MCIc. p > 0.05, not significant.*

The present study included 55 MCI non-converters (MCInc), 30 MCI converters (MCIc), and 19 AD. We divided the MCI patients according to Wolz’s study (Wolz et al., 2011), into MCInc and MCIc, in which MCIc were defined as patients whose diagnosis changed within 36 months and the complementary MCInc patients defined as MCInc group (up to the time of data screening, MCI had not been converted in the database). Also, 19 out of 30 former MCIc developed AD within 36 months (Other 11 subjects were excluded because of the absence of data and data quality control). In the first instance, we took a baseline for all MCI patients. Thereafter, we continued to take scans until the first reported conversion to AD or up to a period of 36 months. As illustrated in Table 1, gender, age, education and CDR had no significant difference for MCInc and AD, compared to the MCIc.

### Data Acquisition and Preprocessing

According to the ADNI acquisition protocol, participants underwent sMRI and rs-fMRI scanning on 3T Philips scanner (Jack et al., 2008). Scan parameters were as follows: sMRI data were acquired with T1-weighted magnetization prepared rapid acquisition gradient echo (MPRAGE) sequences [repetition time (TR) = 3,000 ms; echo time (TE) = 30 ms; matrix = 256 × 256; flip angle = 9°; voxel size = 1.2 mm^3^ × 1.0 mm^3^ × 1.0 mm^3^; 170 slices]. rs-fMRI data were acquired with a gradient echo planar imaging (EPI) sequence (TR = 3,000 ms; TE = 30 ms; matrix = 64 × 64; flip angle = 80°; voxel size = 3.313 mm^3^ × 3.313 mm^3^ × 3.313 mm^3^; 48 slices).

These methods are similar to those used in our previous studies (Wei et al., 2016; Zhang et al., 2019). sMRI data were preprocessed using software FreeSurfer 6.00 (FreeSurfer v6.00)^2^, which contained: automatic Talairach space transformation, correction of the non-uniformity of image intensity, removal of non-brain tissue, intensity normalization, tissue segmentation (Fischl et al., 2002), automatic topology correction, surface deformation to generate gray/white matter boundaries, fragmentation of the gray matter/cerebrospinal fluid boundary, and cerebral cortex. We used the Desikan-Killiany atlas (34 areas in each hemisphere) for parcellation (Desikan Rahul et al., 2006). rs-fMRI data preprocessing was performed using Basic Edition of Data Processing Assistant for Resting-State Functional MR Imaging (DPARSF) (Yan and Zang, 2010), Statistical Parametric Mapping software (Friston et al., 2007) (SPM8)^3^, and Resting-State fMRI Data Analysis Toolkit (Song et al., 2011) (REST)^4^, based on MATLAB 2013a (MathWorks, Inc)^5^ platform, which involved: (1) Discarding of the first 10 time points for signal stabilization. (2) Slice timing. (3) Realigning and limiting head motion to less than 2 mm or 2°. (4) Spatial normalization. (5) Spatial smoothing with FWHM [6 6 6] Gaussian kernel and linear detrending. (6) Regressing out nuisance covariates: white matter (WM), cerebrospinal fluid (CSF) signals, and six head motion parameters (Ciric et al., 2017). (7) The filtering process, here, the low-frequency signal was divided into 0.01–0.08 Hz, 0.027–0.08 Hz and 0.01–0.027 Hz.

### Feature Extraction

**FIGURE 1:**
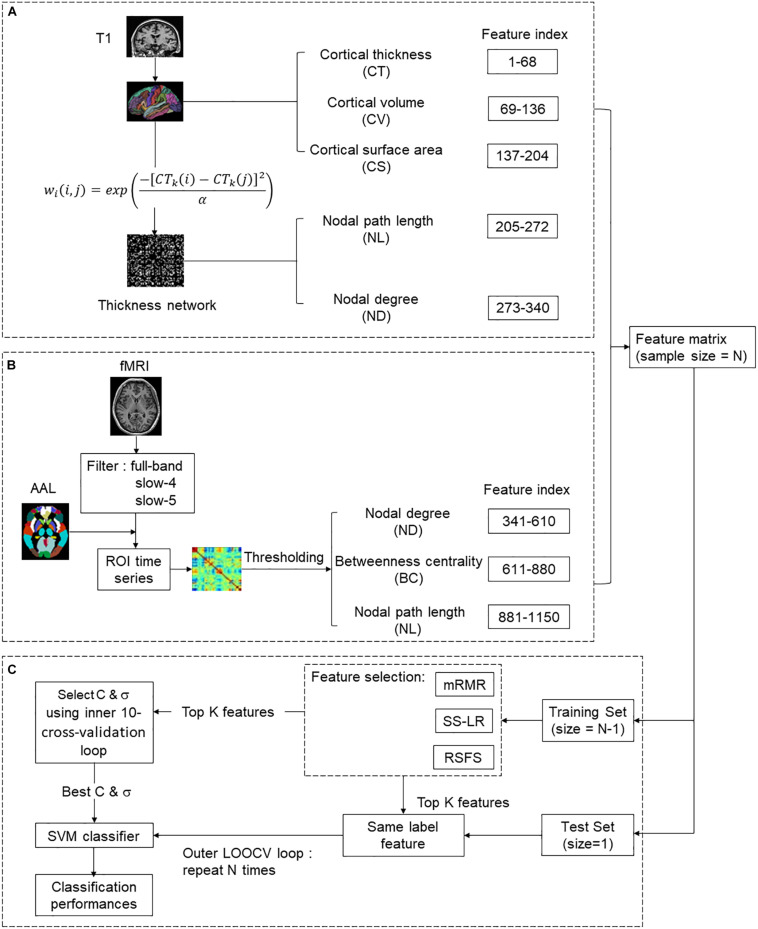
The overall classification framework for predicting the conversion of MCI. **(A)** Structural feature extraction: preprocessing T1 data, extract MRI features and thickness network features. **(B)** Functional feature extraction: preprocessing rs-fMRI data, constructing resting-state functional brain network and extracting features. **(C)** Feature selection and Classification.

As illustrated in Figures 1A,B, we selected 1150 structural and functional features of each subject for subsequent feature selection. (1) In the structural section, there were 68^∗^3 = 204 MRI features [cortical thickness (CT), cortical volume (CV), and cortical surface area (CS)], 68^∗^2 = 136 nodal features [nodal path length (NL) and nodal degree (ND)]. (2) In the functional section, there were 810 nodal features [NL, ND, and betweenness centrality (BC)]. For a given node *i*, *V* is the size of a graph. NL, ND, and BC were defined as follows:

(1) $$L_{i}=\frac{\sum_{j\neq i\in V}L_{ij}}{\left(V-1\right)}$$

where *L*_*ij*_ represents the minimum number of edges between node *i* and *j*,

(2) $$K_{i}=\sum_{j\in V}b_{ij}$$

and *b*_*ij*_ is the connection between node *i* and *j*.

(3) $$B_{i}=\sum_{i\neq j\neq m\in V}\frac{S_{jm}\left(i\right)}{S_{jm}}$$

*S*_*jm*_ represents the number of shortest path lengths between node *m* and *j*, *S*_*jm*_(*i*) represents the number of shortest paths through node *i* between node *m* and *j*.

#### MRI Features

As indicated in Figure 1A, the atlas used in Desikan-Killiany template included 68 cortical regions. For each cortical region, CT, CV, and CS were calculated as MRI features. CT at each vertex of the cortex was defined as the average shortest length between white and pale surfaces. While CV at each vertex was defined as the product of the CS and CT at each surface vertex. On the other hand, CS was defined as a computation of the area of every triangle in a standardized spherical surface tessellation. This section yielded 204 MRI features for each participant.

#### Thickness Network Features

The thickness network matrix *w*_*ij*_ (i, j = 1,2,…,68) was defined by calculating the difference of CT between each pair of regions, as follows:

(4) $$w_{k}\left(i,j\right)=exp\left(\frac{-{\left[CT_{k}\left(i\right)-CT_{k}\left(j\right)\right]}^{2}}{\alpha }\right)$$

Where *CT*_*k*_(*i*) represents the cortical thickness of *i* ROI of *k* participants, and the kernel width α is 0.01. To eliminate the influence of false connections and noise, we thresholded the thickness network matrix of each participant into a binary matrix *B*_*ij*_ = [*b*_*ij*_]. The threshold represents the cost of network connection, defined as the ratio of over-threshold connections to the total number of possible connections in the network (Sanz-Arigita et al., 2010). If the weight of the two ROIs was greater than the given threshold, then *b*_*ij*_ was 1, or otherwise 0. Notably, there is no golden rule for the definition of a single sparsity threshold, and different sparsity will lead to different results (He et al., 2009; Hojjati et al., 2018). Therefore, we analyzed the range of costs from 8 to 44%, at 1% intervals. Finally, 136 nodal features were employed for subsequent analysis (Figure 1A).

#### Functional Network Features

The nodes of the functional brain network were defined by dividing the brain into 90 regions using the automatic anatomical labeling (AAL) template (Tzourio-Mazoyer et al., 2002). The brain network of each participant was a 90^∗^90 connection matrix. Each element of the matrix was the Pearson correlation coefficient between brain regions. Then, we applied Fisher’s r-to-z transformation on the raw undirected connectivity matrix (Wee et al., 2012b). The connection of the brain area itself is meaningless, so the diagonal of the connection matrix was set to zero (Zhan et al., 2013). Consistent with the structural network, we set the threshold 8–44%, at 1% intervals. In this part, 810 nodal features (NL, ND, and BC) were obtained for subsequent feature selection (Figure 1B).

### Feature Selection

In the feature selection section, three feature selection algorithms were applied to classification (Figure 1C).

#### Random Subset Feature Selection Algorithm (RSFS)

The RSFS is an algorithm that can find a set of features whose performance is better than the average feature performance of the available feature set (Pohjalainen et al., 2015). The RSFS process includes the main ideas of the random forest (Breiman, 2001) and random K-Nearest neighbor (KNN) (Li et al., 2011). It repeatedly selects a random feature subset from the set of all possible features and then classifies it by KNN.

In RSFS, *F* represents a full feature set with *j* true features, each true feature *f*_*j*_ from a full set of features *F* has a relevance value *r*_*j*_∈ (-∞, ∞) associated with it. In addition, a set of dummy features *z*_*j*_∈ Z with related relevances *q*_*j*_ is also defined.

During each iteration *i*, the RSFS algorithm mainly executes the following steps:

(1) Randomly select a subset *S*_*i*_ of *n* features (|*S*_*i*_| = *n*) from the full set *F* by sampling from a uniform distribution.

(2) For the given data set, uses *S*_*i*_ to perform KNN classification and calculates the value of the criterion function *c*_*i*_ to measure the performance of classification.

(3) Update *r*_*j*_ of all used *f*_*j*_ by replacing them according to the formula (5):

(5) $$r_{j}^{\prime }=r_{j}+c_{i}-E\left\{c\right\}$$

Where *r*_*j*_ is current relevance value, $r_{j}^{\prime }$ is the updated relevance value, *c*_*i*_ is the current function value and *E*{*c*} is the expectation of the criterion function value (corresponding to the average of all previous iterations of *c*_*i*_). Specifically, relevance (feature indices) = relevance (feature indices) + performance criterion – expected criterion value.

(4) Repeats step (1) with a new random subset.

In parallel to updating the feature relevance, similar processing was performed on virtual features by always selecting a random subset of *m* virtual features and then updating the relevance values of these features according to formula (5) but using the criterion function value of the true features from the same iteration.

Finally, a statistical test was performed to find the feature set *S*⊂ *F*, that truly surpasses the relevance ratings of virtual features. The selection condition formula is as follows:

(6) $$p\left(r_{j}<r_{rand}\right)\geq \delta ,\forall f_{j}\in B,F,$$

In formula (6), *r*_*rand*_ is the baseline level and δ is the probability threshold. The *r*_*rand*_ is modeled as the normal distribution of the virtual correlation *q*_*j*_. Then obtain the probability that the feature is more relevant than a virtual feature from the cumulative normal distribution.

(7) $$p\left(r_{j}<r_{rand}\right)=\frac{1}{\sigma_{\text{g}}\sqrt{2\pi }}\int_{-\infty }^{r_{j}}\exp\left(\frac{-{\left(x-\mu_{\text{g}}\right)}^{2}}{2\sigma_{\text{g}}^{2}}\right)dx$$

Verification was performed in each repeated process of RSFS. If the feature that exceeds the random feature classification performance was no longer selected, the screening was stopped or the feature selection ended by setting a fixed number of program repetitions (Li et al., 2011; Pohjalainen et al., 2015).

#### Minimal Redundancy Maximal Relevance Feature Selection Algorithm (mRMR)

We used mRMR proposed by Ding and Peng for feature selection (Peng et al., 2005). mRMR can use mutual information as a measure to solve the trade-off between feature redundancy and relevance (Morgado and Silveira, 2015).

Max-Relevance is defined as:

(8) $$\max D\left(S,c\right),D=\frac{1}{\left|S\right|}\sum_{x_{i}\in S}I\left(x_{i};c\right)$$

*S* represents a feature set with *m* features {*x*_*i*_}, *D* is the mutual information value between the attribute subset, and the label and *c* is the class.

Min-Redundancy is defined as:

(9) $$\min R\left(S\right),R=\frac{1}{{\left|S\right|}^{2}}\sum_{x_{i},x_{j}\in S}I\left(x_{i},x_{j}\right)$$

*R* represents the mutual information value between feature attributes.

The combination of formula (8) and formula (9) is the criterion for selecting feature subsets with minimum redundancy and maximum relevance. Therefore, mRMR was defined as:

(10) $$mRMR=\max_{S}\left\{\frac{1}{\left|S\right|}\sum_{x_{i}\in S}I\left(x_{i};c\right)-\frac{1}{{\left|S\right|}^{2}}\sum_{x_{i},x_{j}\in S}I\left(x_{i},x_{j}\right)\right\}$$

#### Sparse Linear Regression Feature Selection Algorithm Based on Stationary Selection (SS-LR)

The SLEP package (Liu et al., 2009) was used to solve sparse linear regression. Given a data set *T* = (*X*, *Y*), where *X* = (*x*_1_, *x*_2_, …, *x*_*n*_)^*T*^ ∈ *R*^*n*×*m*^ is the sample, *Y* = (*y*_1_, *y*_2_, …, *y*_*n*_)^*T*^ ∈ *R*^*n*×1^ is a true label, *n* is the number of samples, and *m* is the number of features for each sample. The linear regression model can be defined as:

(11) f(X)=Xw

Where the coefficient of the linear regression is defined as *w* = (*w*_1_, *w*_2_, …, *w*_*n*_) ∈ *R*^*m*×1^, *f*(*X*) is the predicted label vector obtained by distinguishing the unknown samples. Let *L*(*w*) be the loss function of linear regression, the function is defined as a formula (12):

(12) $$L\left(w\right)=\min_{w}\frac{1}{n}{||f\left(X\right)-Y||}_{2}^{2}$$

Add an *L*_1_ regularization term after the loss function to control the complexity of the model, and add the regularized expression:

(13) $$L\left(w\right)=\min_{w}\frac{1}{n}{||f\left(X\right)-Y||}_{2}^{2}+\lambda {||w||}_{1}$$

Where ||*w*||_1_ = $\sum_{i=1}^{m}\left|w_{i}\right|$, λ > 0 is the regularization parameter of the model control. As λ increases, the sparseness of the function becomes larger. The range is 0.05 < λ < 0.3 and the step size is 0.005. Sub-sampling or bootstrapping from the original sample for stability selection to solve the problem of proper regularization (Meinshausen and Bühlmann, 2010).

### SVM Classifier

The SVM classifier adopted here comes from the LIBSVM software package, which was developed by Lin’s team (Chang and Lin, 2011). The kernel function in the SVM classifier uses the radial basis kernel function (RBF), where the penalty parameter 𝒞 and the kernel bandwidth σ in the kernel function range from [4^−4^, 4^4^]. The RBF kernel was defined as follows:

(14) $$K\left(X_{1},X_{2}\right)=exp\left(-\frac{||X_{1}-X_{2}||}{2\sigma^{2}}\right)$$

where *X*_1_, *X*_2_ are two eigenvectors, σ is the width parameter of the REF kernel. Both internal and external cross-validation methods were used in Figure 1C. Internal cross-validation was used to find the best classifier parameters, and external cross-validation was used to verify the performance of the classifier. A nested cross-validation was used to obtain unbiased estimates. After normalization and feature screening of the training data set, an internal cross-validation (10-fold cross-validation and grid search method) was performed on the training set (inner loop). In the outer loop, leave-one-out cross-validation (LOOCV) was repeated for N (N = 85 or 49) times. Finally, the held-out sample was used to evaluate the training classifier. These parameters were defined as follows (Fawcett, 2006; Wee et al., 2012b):

$$Accurary=\frac{TP+TN}{TP+TN+FP+FN},$$

(15) $$Sensitivity=\frac{TP}{TP+FN},Specificity=\frac{TN}{TN+FP},$$

(16) $$BalancedAccuracy\left(BAC\right)=\frac{Sensitivity+specificity}{2}$$

where TP is true positive; TN, true negative; FP, false positive and FN, false negative respectively. Area Under Curve (AUC) was defined as the area under the ROC curve and the coordinate axis.

### Statistical Analysis

All statistical calculations were performed in the matlab2016b platform (MathWorks, Inc, see text footnote 5). The exact Clopper–Pearson method was used to calculate the 95% confidence intervals (CIs) of sensitivity, specificity, and accuracy (Agresti and Coull, 1998). The CIs of AUC was calculated by the DeLong methods (DeLong et al., 1988; Mercaldo et al., 2007; Mei et al., 2020). McNemar’s test (Bates and McNemar, 1964) was used to calculate the two-sided *P*-value for AUC between MCInc vs. MCIc, AD vs. MCIc.

## Results

### Classification Results

**FIGURE 2:**
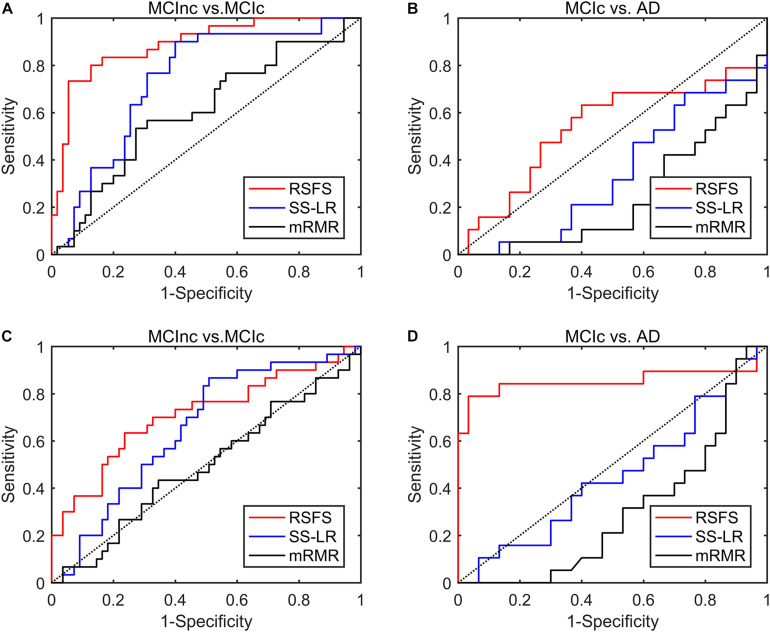
ROC curves of the three algorithms performed SVM classifier using the top 10 features. **(A)** MCInc vs. MCIc group at cost = 39%, **(B)** MCIc vs. AD group at cost = 39%. **(C)** MCInc vs. MCIc group at cost = 19%, **(D)** MCIc vs. AD group at cost = 19%.

To reduce feature redundancy for each threshold containing 1150 features, the features of the two classification groups (MCInc vs. MCIc, MCIc vs. AD) were selected by the RSFS, SS-LR, and mRMR in the cost range of 8–44%. The classification results showed that the AUC and ACC obtained by the RSFS algorithm were significantly higher than the other algorithms (Supplementary Figures 1A,B). By comparison, it was found that the classification result obtained by the MCInc vs. MCIc group at cost = 39%, was the best and the most stable, and the classification result obtained by the MCIc vs. AD group at cost = 19%, was the best and the most stable. Therefore, the subsequent results were analyzed and discussed in cost = 39 and 19%. The receiver operating characteristic (ROC) curves and classification results are depicted in Figure 2 and Table 2.

**TABLE 2:** Classification results performance of different methods using the top 10 features.

GROUP	RSFS				SS-LR				mRMR			

	ACC (%)	SEN (%)	SPE (%)	AUC	ACC (%)	SEN (%)	SPE (%)	AUC	ACC (%)	SEN (%)	SPE (%)	AUC
MCInc vs. MCIc	84.71 [75.27, 91.60]	66.67 [47.19, 82.71]	94.55 [84.88, 98.86]	0.888 [0.814, 0.962]	65.88 [54.80, 75.82]	50.00 [31.30, 68.70]	74.55 [61.00, 85.33]	0.738 [0.629, 0.847]	61.18 [49.99, 71.56]	33.33 [17.29, 52.81]	76.36 [62.98, 86.77]	0.605 [0.478, 0.733]
MCIc vs. AD	89.80 [77.77, 96.60]	78.95 [54.43, 93.95]	96.67 [82.78, 99.92]	0.854 [0.709, 1.000]	51.02 [36.34, 65.58]	36.84 [16.29, 61.64]	60.00 [40.60, 77.34]	0.451 [0.281, 0.620]	40.82 [27.00, 55.79]	5.26 [0.13, 26.03]	63.33 [43.86, 80.07]	0.297 [0.151, 0.444]

*ACC, accuracy; SEN, sensitivity; SPE, specificity; AUC, area under the curve. AUC comparisons were evaluated by the DeLong test to compute the 95% CI; accuracy, sensitivity and specificity comparisons were calculated by using the exact Clopper–Pearson method to compute the 95% CI; all CIs shown in parentheses.*

In MCInc vs. MCIc group, the RSFS algorithm achieved an 84.71% accuracy (95% CI 75.3%, 91.6%), an 66.67% sensitivity (95% CI 47.2%, 82.7%), a 94.55% specificity (95% CI 84.9%, 98.9%) and 0.888 AUC (95% CI 0.814, 0.962). The SS-LR algorithm had an 65.88% accuracy (95% CI 54.80%, 75.82%), 50.0% sensitivity (95% CI 31.30%, 68.70%), 74.55% specificity (95% CI 61.00%, 85.33%), and 0.738 AUC (95% CI 0.629, 0.847). The mRMR algorithm had 61.18% accuracy (95% CI 49.99%, 71.56%), 33.33% sensitivity (95% CI 17.29%, 52.81%), 76.36% specificity (95% CI 62.98%, 86.77%), and 0.605 AUC (95% CI 0.478, 0.733).

In MCIc vs. AD group, the RSFS algorithm achieved an 89.80% accuracy (95% CI 77.77%, 96.60%), 78.95% sensitivity (95% CI 54.43%, 93.95%), 96.67% specificity (95% CI 82.78%, 99.92%), 0.854 AUC (95% CI 0.709, 1.000). The SS-LR algorithm had 51.02% accuracy (95% CI 36.34, 65.58), 36.84% sensitivity (95% CI 16.29, 61.64), 60.00% specificity (95% CI 40.60, 77.34) and 0.451 AUC (95% CI 0.281, 0.620). The mRMR algorithm had 40.82% accuracy (95% CI 27.00, 55.79), 5.26% sensitivity (95% CI 0.13, 26.03), 63.33% specificity (95% CI 43.86, 80.07), and 0.297 AUC (95% CI 0.151, 0.444).

#### Comparing Classification Results Based on Different Feature Selection Methods

**FIGURE 3:**
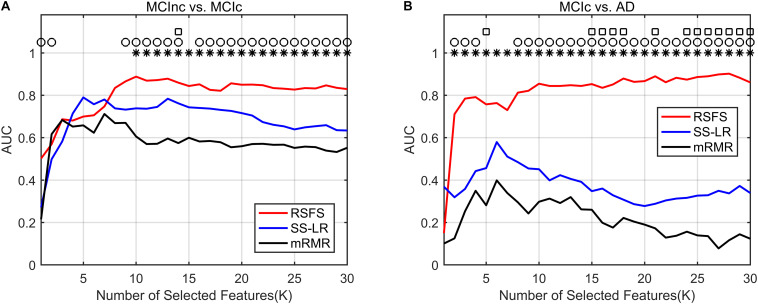
Comparison of AUC scores of three algorithms performed SVM classifier. Subgraphs **(A)** and **(B)** represent AUC scores with the number of features K of MCInc vs. MCIc, and MCIc vs. AD. ○ Indicate the classification performance of the RSFS algorithm and SS-LR algorithm is significantly different. 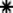 Indicate the classification performance of the RSFS algorithm and mRMR algorithm is significantly different. □ Indicate the classification performance of the mRMR algorithm and SS-LR algorithm is significantly different.

In Figure 3, the top K features (K = 1, 2,…, 30) were used for classification to prove the effect of the number of selected features on the classification performance respectively. After the top 8 features, the AUC curves appeared stable in the two groups. In MCIc vs. AD group, the AUC curves of the mRMR algorithm and SS-LR algorithm go downward and can hardly be classified correctly. We compared the classification performance of the three feature selection algorithms, and the results are shown in Table 3 and Figure 3. As shown in Table 3, the classification performance obtained by the RSFS algorithm showed significant differences compared to those obtained by the mRMR algorithm and the FS algorithm in the two classification groups. But we found no significant difference between the mRMR algorithm and the FS algorithm.

**TABLE 3:** Comparison of classification results between different feature selection methods.

GROUP	Sig.(RSFS vs. SS-LR)	Sig.(RSFS vs. mRMR)	Sig.(mRMR vs. SS-LR)
MCInc vs. MCIc	0.001383	0.000329	0.479500
MCIc vs. AD	0.000085	0.000006	0.358795

*The “Sig.” column gives the p-value. McNemar’s test to calculate the p-value.*

As illustrated in Figure 3A, the AUC scores of the RSFS algorithm were significantly higher than those of the SS-LR algorithm (K = 1, 2, 9–14, 16–30) and mRMR algorithm (K = 10–30) in MCInc vs. MCIc group. At K = 14, the AUC scores of the three algorithms showed significant differences. As shown in Figure 3B, the AUC scores of the RSFS algorithm were significantly higher than those of the SS-LR algorithm (K = 2–4, 8–30) and mRMR algorithm (K = 2–30) in MCIc vs. AD group. At K = 5, 15–18, 21, 24–30, the AUC scores obtained by the SS-LR algorithm were significantly higher than those obtained by mRMR. We found that the AUC scores of the three algorithms have significant differences (K = 15–18, 21, 24–30).

In summary, the classification results of the RSFS algorithm in the MCInc vs. MCIc group was the best, followed by that of the SS-LR algorithm, and then the mRMR algorithm. For the MCIc vs. AD group, the classification results of the RSFS algorithm was also the best, while the classification results obtained by using the other algorithms were relatively poor. Hence, only the two classification groups of results obtained by applying the RSFS algorithm are discussed below.

#### Confirmatory Analyses – Further Resampling Results

With the higher AUC and ACC, the classification effect obtained by the RSFS algorithm outperformed the SS-LR algorithm and mRMR algorithm (Figure 2 and Table 2). In Table 2, it is observable that the imbalanced data caused a gap between sensitivities and specificities. Therefore, we compared the performance of multiple classifiers and verified the reliability of our results through upsampling. As shown in Table 4 and Supplementary Figure 2, the upsampled data were trained and tested by four classifiers (Random Forest (Breiman, 2001), KNN (Yang et al., 2007), AdaBoost (Hastie et al., 2009), SVM). The results showed that the classification accuracy obtained by SVM was the highest and equally matched the results before upsampling.

**TABLE 4:** Classification performance of multiple classifiers based on RSFS algorithm using the top 10 features.

	MCInc vs. MCIc				MCIc vs. AD			
	**ACC(%)**	**SEN(%)**	**SPE(%)**	**AUC**	**ACC(%)**	**SEN(%)**	**SPE(%)**	**AUC**
RF^*a*^	67.06	46.67	78.18	0.742	67.35	57.89	73.33	0.716
KNN^*a*^	69.41	93.33	56.36	0.887	63.27	84.21	50.00	0.884
Adaboost^*a*^	69.41	60.00	74.55	0.725	71.43	52.63	83.33	0.763
SVM^*a*^	84.71	83.33	85.45	0.886	87.76	73.68	96.67	0.849
SVM^*b*^	84.71	66.67	94.55	0.888	89.80	78.95	96.67	0.854

*ACC, accuracy; SEN, sensitivity; SPE, specificity. RF, Random Forest; KNN, k-nearest neighbor classification. ^a^Represents the use of upsampling balanced data for classification. ^b^Represents the use of original data for classification.*

The reported results of this study were based on only a limited number of iterations (based on the number of subjects) which may be the main reason for the high classification performances. To address this issue and considering the impact of single sampling on classification performance, we upsampling and downsampling the data (Dubey et al., 2013; Hojjati et al., 2017). In general, we performed 500 iterations of the outer loop in the resampling part, and performed the leave-one-out method in the inner loop (For upsampling, based on the number of samples in MCIc vs. AD group is 60 or the number of samples in MCInc vs. MCIc group is 110) for classification prediction, and finally reported the average of those performances average ((60 or 110) × 500 iterations) as the classification result. As illustrated in Supplementary Figures 3, 4, these results show that the result classification performance of the original nosampling data is between upsampling and downsampling when the number of features is 1–30. We compared the classification performance of resample data based on RSFS algorithm and SVM classifier using the top 10 features, and the results are shown in Table 5. In MCInc vs. MCIc group, compare with classification performance of the downsampling (80.20% accuracy, 76.37% sensitivity, 84.03% specificity, 0.853 AUC), nosampling classification performance were slightly higher. However, upsampling classification performance were greater than 90%. In MCIc vs. AD group, compare with classification performance of the downsampling (80.80% accuracy, 71.87% sensitivity, 89.73% specificity, 0.827 AUC), nosampling classification performance were slightly higher, upsampling1 classification performance were greater than those of nosampling. But the accuracy of upsampling2 was lower than that of nosampling. Based on the above results, this study analyzed and compared the nosampling data in the following analysis.

**TABLE 5:** Classification performance of resample data based on RSFS algorithm and SVM classifier using the top 10 features.

	MCInc vs. MCIc				MCIc vs. AD			
	**ACC(%)**	**SEN(%)**	**SPE(%)**	**AUC**	**ACC(%)**	**SEN(%)**	**SPE(%)**	**AUC**
nosampling	84.71	66.67	94.55	0.888	89.80	78.95	96.67	0.854
downsampling	80.20	76.37	84.03	0.853	80.80	71.87	89.73	0.827
upsampling1	91.59	90.00	93.18	0.953	91.57	85.65	97.49	0.947
upsampling2	92.70	91.74	93.66	0.962	88.90	80.59	97.21	0.934

*Downsampling and upsampling1 are defined as random resampling. Upsampling2 is defined as ensuring that each original sample is included, and then randomly resampling the remaining data.*

#### Highly Sensitive Characteristic

**FIGURE 4:**
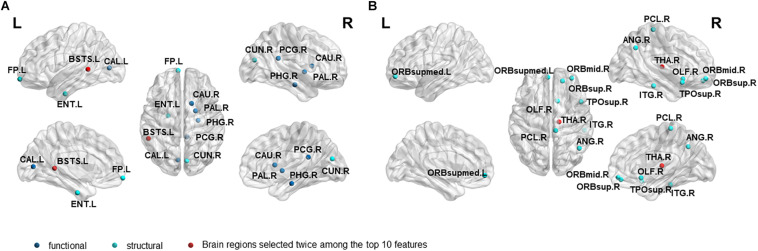
The location and networks attribution of top 10 brain regions, listed in Tables 6 **(A)**, 7 **(B)**, which might be affected in early stage of MCI.

In order to investigate which features are highly sensitive brain regions related to MCI disease, we accumulate the number of selected features used for classification, and finally obtain the frequency of occurrence of all selected features. Tables 6, 7 and Figure 4 summarize the details of the top 10 features that can be used to distinguish MCInc and MCIc, MCIc and AD. As shown in Table 6, there was 30% structural features, 20% structural connectivity network features, and 50% functional connectivity network features. Consistent with the previous studies, the brain regions selected by our method to identify MCInc subjects from MCI included the left banks superior temporal sulcus (Khazaee et al., 2017), left entorhinal cortex (Zhang et al., 2011; Nickl-Jockschat et al., 2012; Suk et al., 2015; Rasero et al., 2017), right caudate nucleus (Khazaee et al., 2015; Suk et al., 2015), left calcarine fissure and surrounding cortex (Khazaee et al., 2015; Wang et al., 2015; Pusil et al., 2019), left frontal pole (Wee et al., 2014), right parahippocampal gyrus (Suk et al., 2015; Hojjati et al., 2017; Pusil et al., 2019), right lenticular nucleus, pallidum (Zhang et al., 2011), right cuneus cortex (Nickl-Jockschat et al., 2012; Suk et al., 2015), right posterior cingulate gyrus (Khazaee et al., 2015).

**TABLE 6:** Selected feature distributions in the MCInc vs. MCIc group using the RSFS algorithm.

Feature index	Modality	Frequency band	Networks attribution	Region	Frequency (%)
1	Structural		Thickness	BSTS.L	100
39	Structural		Thickness	ENT.L	100
862	FCN	Slow-4	BC	CAU.R	100
1013	FCN	Slow-5	ND	CAL.L	100
337	SCN		NL	FP.L	98.82
830	FCN	Slow-4	BC	PHG.R	98.82
506	FCN	Full band	ND	PAL.R	97.65
208	SCN		ND	CUN.R	94.12
69	Structural		Volume	BSTS.L	92.94
826	FCN	Slow-4	BC	PCG.R	36.47

*FCN, functional connectivity network; SCN, structural connectivity network; BSTS.L, Left Banks superior temporal sulcus; ENT.L, Left Entorhinal cortex; CAU.R, Right Caudate nucleus; CAL.L, Left Calcarine fissure and surrounding cortex; FP.L, Left Frontal pole; PHG.R, Right Parahippocampal gyrus; PAL.R, Right Lenticular nucleus, pallidum; CUN.R, Right Cuneus cortex; PCG.R, Right Posterior cingulate gyrus.*

**TABLE 7:** Selected feature distributions in the MCIc vs. AD group using the RSFS algorithm.

Feature index	Modality	Frequency band	Networks attribution	Region	Frequency (%)
440	FCN	Full band	ND	ORBmid.R	100
778	FCN	Slow-4	ND	THA.R	100
1066	FCN	Slow-5	BC	ORBsup.R	100
1082	FCN	Slow-5	BC	OLF.R	100
688	FCN	Slow-4	NL	THA.R	87.76
1126	FCN	Slow-5	BC	ANG.R	85.71
590	FCN	Full band	BC	PCL.R	73.47
880	FCN	slow-4	BC	ITG.R	71.43
874	FCN	Slow-4	BC	TPOsup.R	46.94
545	FCN	Full band	BC	ORBsupmed.L	44.90

*FCN, functional connectivity network; ORBmid.R, Right Middle frontal gyrus orbital part; THA.R, Right Thalamus; ORBsup.R, Right Superior frontal gyrus, orbital part; OLF.R, Right Olfactory cortex; ANG.R, Right Angular gyrus; PCL.R, Right Paracentral lobule; ITG.R, Right Inferior temporal gyrus; TPOsup.R, Right Temporal pole, superior temporal gyrus; ORBsupmed.L, Left Superior frontal gyrus, medial orbital.*

As demonstrated in Table 7, all features came from the functional network and the proportion of the three frequency bands is 3(full-band):3(slow-5):4(slow-4). Moreover, it should be noted that 70% of features came from betweenness centrality. The selected brain regions included the right middle frontal gyrus orbital part (Khazaee et al., 2015), right thalamus (Nickl-Jockschat et al., 2012; Khazaee et al., 2015), right superior frontal gyrus, orbital part (Suk and Shen, 2014), right olfactory cortex (Khazaee et al., 2015), right angular gyrus (Suk et al., 2015; Wang et al., 2015),right paracentral lobule (Suk and Shen, 2014), right inferior temporal gyrus (Wee et al., 2014), right temporal pole: superior temporal gyrus (Wee et al., 2014; Khazaee et al., 2015), left superior frontal gyrus, and medial orbital (Khazaee et al., 2015; Wang et al., 2015; Pusil et al., 2019).

## Discussion

In the present study, we used structure-functional MRI and the combined graph theory with multiple machine learning methods to accurately classify patients with MCIc and MCInc/AD. Our findings demonstrated that, by including the cortical thickness features, structural brain network features, and sub-frequency (full-band, slow-4, slow-5) functional brain network features, the proposed method performed effectively in identifying MCIc subjects from MCInc/AD. In the classifications of MCIc vs. MCInc and MCIc vs. AD, the proposed RSFS algorithm achieved the best accuracies (84.71%, 89.80%) compared to other algorithms (Table 2 and Figure 3).

In Table 2, there is a gap between specificities and sensitivities due to the imbalanced data. However, our proposed method obtained the best BAC of 80.61 and 87.81% with the RSFS algorithm. We also compared the performance of multiple classifiers and verified the reliability of our results through upsampling (Supplementary Figure 2). The results indicated that the SVM classifier obtained the best accuracy, and was consistent with the results before upsampling. The balance of sensitivities and specificities has also been appropriately improved. In addition, we observed that the mRMR algorithm achieved 5.26% sensitivity in MCIc vs. AD group compared to other methods as described in Table 2. Actually, as shown in Supplementary Figures 1A,B, the SS-LR algorithm and the mRMR algorithm achieved best performance (84.71% ACC, 73.33% SEN, 90.91% SPE, 83.45% AUC at cost = 27%, K = 4 and 77.65% ACC, 53.33% SEN, 90.91% SPE, 74.45% AUC at cost = 8%, K = 20, respectively) in MCInc vs. MCIc group. The SS-LR algorithm and the mRMR algorithm achieved the best performance (71.43% ACC, 42.11% SEN, 90.00% SPE, 70.53% AUC at cost = 36%, K = 2 and 71.43% ACC, 52.63% SEN, 83.33% SPE, 70.35% AUC at cost = 33%, K = 12, respectively) in MCIc vs. AD group.

As illustrated in Tables 8, 9, the classification results obtained by the combination of sMRI and rs-fMRI in the present study are better than those of the unimodal (sMRI\rs-fMRI) approach, including those of our previous studies (Wei et al., 2016; Zhang et al., 2019). Meanwhile, we also compared the classification performances with other studies. Most previous methods that constructed brain networks only considered structural or functional features (Suk and Shen, 2014; Hu et al., 2015; Moradi et al., 2015; Raamana et al., 2015; Ardekani et al., 2016; Suk et al., 2016; Beheshti et al., 2017; Hojjati et al., 2017, 2018; Zheng et al., 2019; Gupta et al., 2020; Zhu et al., 2021), and obtained an accuracy lower than that of the present study. Only Hojjatia’s study (Hojjati et al., 2017) used graph theory and machine learning approach (mRMR, FS) to classify rs-fMRI and obtained a classification accuracy of 91.4%. However, the sample size was too small (<20), and the effect was not widely representative. Besides, the studies in Table 8, Zhang and Shen (2012) used a multi-modal multi-task learning algorithm to fuse MRI, FDG-PET, and CSF data and regressed the MMSE and ADAS-Cog scores to classify MCInc and MCIc with a classification accuracy of 73.9%. Similarly, Cui et al. (2011) combined MRI, CSF, and cognitive scoring scale features to classify MCInc and MCIc with a classification accuracy of 67.13%. Ye et al. (2012) used sMRI, ApoE, and cognitive scores to classify MCIc and MCInc using a smooth selection method based on sparse logistic regression, and obtained good classification results of 0.859 AUC. Therefore, these results may suggest that the method we have proposed could effectively help predict the conversion to Alzheimer’s disease.

**TABLE 8:** Classification performance of different methods to distinguish different stages of MCI.

Article	Method	Cohort	ACC (%)	SEN (%)	SPE (%)	AUC
Proposed	rs-fMRI, sMRI, graph theory, machine learning approach (RSFS)	MCIc/MCInc (30/55)	84.71	66.67	94.55	0.888
	sMRI, graph theory, RSFS	MCIc/MCInc	68.24	80.00	46.67	0.673
	rs-fMRI, graph theory, RSFS	MCIc/MCInc	64.71	78.18	40.00	0.670
Wei et al., 2016	Combination of MRI and thickness network (SS-LR)	MCIc/MCInc (61/83)	76.40	65.60	84.30	0.813
Hojjati et al., 2017	rs-fMRI, graph theory, machine learning approach (mRMR, FS)	MCIc/MCInc (18/62)	91.40	83.24	90.10	N/A
Hojjati et al., 2018	rs-fMRI, sMRI, 6 features, graph theory, machine learning approach (mRMR)	MCIc/MCInc (18/62)	97.00	95.00	100	0.980
Suk and Shen, 2014	93 features from a MR image and the same dimensional features from a FDG-PET image.	MCIc/MCInc (43/56)	74.04	58.00	82.67	0.696
Suk et al., 2016	MRI, DW-S^2^MTL	pMCI/sMCI (43/56)	69.84	44.00	89.00	N/A
Moradi et al., 2015	MRI, age and cognitive measures 10-fold cross-validation	pMCI/sMCI (164/100)	81.72	86.65	73.64	0.902
Raamana et al., 2015	Thickness network fusion	MCIc/MCInc (56/130)	64.00	65.00	64.00	0.680
Hu et al., 2015	sMRI, tight wavelet frame, SVM	MCIc/MCInc (71/62)	76.69	71.83	82.26	0.790
Ardekani et al., 2016	hippocampal volumetric integrity (HVI) from structural MRI scans RF with 5,000 trees	pMCI/sMCI (86/78)	82.30	86.00	78.20	N/A
Beheshti et al., 2017	sMRI, t-test scores and a genetic algorithm, SVM	pMCI/sMCI (71/65)	75.00	76.92	73.23	0.751
Zheng et al., 2019	MRI and FDG-PET, PCA, SVM	pMCI/sMCI (51/75)	79.37	74.51	82.67	0.892
Gupta et al., 2020	sMRI, FDG-PET, AV45-PET, rs-fMRI, DTI and APOE genotype, MKL	MCIc/MCInc (31/30)	95.08	100	93.93	0.969
Zhu et al., 2021	sMRI, patch-level features, DA-MIDL	pMCI/sMCI (172/232)	80.20	77.10	82.60	0.851

*The best multivariate predictors of MCI conversion are shown for each study.ACC, accuracy; SEN, sensitivity; SPE, specificity; AUC, area under the curve; pMCI, progressive MCI; sMCI, stable MCI; FDG-PET, fluorideoxyglucose positron emission tomography; RF, Random forest; DW-S^2^MTL, deep weighted subclass-based sparse multi-task learning; PCA, principal component analysis. MKL, multiple kernel learning. DA-MIDL, dual attention multi-instance deep learning network.*

**TABLE 9:** Classification of MCIc and AD.

Article	Method	Cohort	ACC (%)	SEN (%)	SPE (%)	AUC
Proposed	rs-fMRI, sMRI, graph theory, machine learning approach (RSFS)	MCIc/AD (30/19)	89.80	78.95	96.67	0.854
	sMRI, graph theory, RSFS		57.14	15.79	83.33	0.428
	rs-fMRI, graph theory, RSFS		77.55	63.16	86.67	0.812

Different from the previous studies, our research not only focused on the brain regions’ conversion sensitivity of the two groups of patients (MCIc vs. MCInc), but also studied the conversion sensitivity of the brain regions of the same group of patients (MCIc vs. AD). Tables 6, 7 and Figure 4 list the highly sensitive brain regions selected from the two groups. These results proved the inconsistency of the selected brain regions in the two classification groups. As shown in Table 6, there were 30% structural features, 20% structural connectivity network features, 50% functional connectivity network features. The proportion of functional connectivity network features in each frequency band is listed as follows: 1(full-band):1(slow-5):3(slow-4). In Table 7, all features came from the functional network and the proportion of the three frequency bands was 3(full-band):3(slow-5):4(slow-4). Moreover, it is worth noting that 70% of features came from betweenness centrality. Our results suggest that the betweenness centrality in a functional network carries more disease information and the top 10 selected features are more sensitive to more efficient classification for MCIc and AD. According to Tables 6, 7, it can be seen that the network parameter characteristics of all frequency bands from rs-fMRI have been selected. However, the cortical surface area (CS) was not selected for the top 10 features in two classification groups by three algorithms. More importantly, in Wei’s work (Wei et al., 2016), the selected top 10 combined structure features did not include CS. Based on the above results, we consider that CS is not an effective marker for AD disease. In future work, we will assess whether it can be excluded from the feature set. Different from our previous work on EMCI and LMCI classification (Zhang et al., 2019) the characteristics of the slow-5 band did not show high sensitivity in MCInc and MCIc classification. The reason may be that the former is mainly based on the degree of memory impairment of MCI disease, and the latter is based on the longitudinal time diagnosis status to classify whether MCI develops into AD. Therefore, we suggest that the difference in their brain activity may be reflected in different frequency bands.

Our findings converge nicely with what has been suggested by the previous studies (see Results Section), and these selected brain regions have been shown to be related to MCI conversion. The important roles of several brain regions in MCI disease have been widely recognized. Braak and Braak (1991) used structural magnetic resonance imaging (sMRI) to study AD patients. They first discovered a large number of neurofibrillary tangles in the medial temporal lobe, and the brain areas involved mainly included the olfactory cortex, hippocampus, and parahippocampal gyrus, amygdala, and cingulate cortex area, which is consistent with the conclusion that the brain atrophy of AD or MCI patients are mainly located in the medial temporal lobe (Fan et al., 2008; Das et al., 2015). In line with the previous studies (Khazaee et al., 2015; Wang et al., 2015; Pusil et al., 2019), we also found that the left calcarine fissure and the surrounding cortex are associated with MCI conversion to AD. Damage to this brain area may cause central visual diseases (such as macular avoidance and hallucinations). Studies have reported that visual impairment can affect patients’ cognition, thereby increasing the risk of dementia (Uhlmann et al., 1991; Naël et al., 2019). Besides, the top 10 highly sensitive features provided by the other two algorithms are also listed in the Supplementary Material (Supplementary Tables 3.1–3.4). Although the sensitivity was lower than that of the RSFS algorithm, the selected top 10 highly sensitive features are also important to brain areas related to AD disease. It shows that the classification framework of graph theory and machine learning methods considering structural and functional MRI provides a new view for improving MCI clinical prediction and diagnosis. Moreover, our findings suggest that the inconsistency of the selected brain regions between the two classification groups requires more attention. The transformation of MCI disease may imply that the structure of the brain area changed in the early stage of AD, and the function of the brain area later began to degenerate. Inconsistency of the brain regions obtained by the two classification groups indicates that the conversion sensitivity brain regions of the two group patients (MCInc vs. MCIc) and the same group patients (MCIc vs. AD) may be different, which further suggests that the classification between the different groups of patients provides limited information. For the follow-up within a group, it may be more meaningful for the study of diseases.

In the current study, the best performances achieved with costs of 39 and 19% based on MCInc vs MCIc group MCIc vs. AD group, respectively. The cost was defined as the ratio of the number of above-threshold edges to the total number of edges in a network. Cost range can be defined from 0 to 1, but the upper limit is generally less than 50% (Tan et al., 2019). Compared to cost = 19%, cost = 39% is the low threshold. Compared to the MCIc vs. AD group, the MCInc vs. MCIc group can be distinguished when the cost is large and there are more edges in the network. Refer to the study of Jie et al. (2014), as the threshold increases, weak connections and unimportant connections are removed, and significant differences are found between different groups of patients. Therefore, we suggested that the best classification performance of the two classification groups at different costs is due to the different topological properties of the brain network. Specifically, the larger the cost, the higher the global and local efficiency, the higher the clustering coefficient, the lower the characteristic path length, and the lower the small-world attributes (Zhang et al., 2019). The difference between brain network parameters is significant, and the topological characteristics of brain regions can be better distinguished. In the future, we will investigate the specific differences in the brain network characteristics of different groups of patients, and combine their clinical scales for predictive analysis.

However, this study has several limitations. One major limitation is the small sample size. Another limitation is the imbalanced data. Despite the promising results of using the RSFS algorithm and the SVM to screen patients with MCIc, further data collection is required to test the generalizability of the method to other patient populations. In future studies, a larger sample should be collected, and the number of subjects balanced as the scale of the ADNI database is expanding (Aisen et al., 2010). Furthermore, future studies should attempt to explore different methods of classification in different stages of AD, including the interpretability of structural and functional brain abnormalities (Ibrahim et al., 2021). The versatility in multiple data sets will be necessary to validate the robustness of the models. For the study of the topological properties of the brain, Power-264 brain regions might be considered as a template for constructing brain networks. In addition, other well-known prognostic information (DTI, ApoE status, Tau/Amyloid/FDG-PET) will be considered for classification (Gupta et al., 2020; Fan et al., 2021). In terms of subject design, we believe that the follow-up data within the subject can better reveal the brain area where the sensitive characteristics of the transformed biomarker are located. The limitation is that the data sample size is too small. If there are subjects who can collect follow-up data through cognitive training (Hernes et al., 2021) and set a baseline control at the same time, more meaningful and reliable results may be obtained.

## Conclusion

The present study investigated the predictive power of cortical thickness features and brain connectivity network features derived from the sMRI and rs-fMRI to identify individuals with MCI from MCInc/AD for the first time. For the selection of subjects, we proposed a mixed-subject method with an inter- (horizontal) and intra-subject design (longitudinal, follow up), which is rarely used in AD classification. In this classification framework, multiple modalities integration was achieved by using graph theory and machine learning algorithms. We found that this framework improves the classification performance of identifying precursor AD (MCIc), and the high-sensitivity features derived with two classification groups are inconsistent. These findings indicate that the converted sensitivity brain regions of the two groups of patients (MCInc vs. MCIc) and the same group of patients (MCIc vs. AD) may be different, which further indicates that the former way of classifying two different groups of patients may provide limited information. Ultimately, such a classification framework integrating information from sMRI and fMRI can effectively predict the conversion of MCI, and different brain regions obtained in this framework from inter-subject and intra-subject design are probably diagnostic markers for AD.

## Data Availability Statement

Publicly available datasets were analyzed in this study. This data can be found here: adni.loni.usc.edu.

## Ethics Statement

The studies involving human participants were reviewed and approved by Ethics approval and informed consents were obtained from the Alzheimer’s Disease Neuroimaging Initiative (ADNI). The patients/participants provided their written informed consent to participate in this study.

## Author Contributions

TZ: roles/writing – original draft, conceptualization, investigation, methodology, writing – review and editing. QL: resources. DZ: investigation. CZ: software. JY and RN: writing – review and editing. JZ and ZJ: funding acquisition and writing – review and editing. LL: conceptualization, supervision, funding acquisition, project administration, and writing – review and editing. All authors reviewed the manuscript.

## Conflict of Interest

The authors declare that the research was conducted in the absence of any commercial or financial relationships that could be construed as a potential conflict of interest.

## Publisher’s Note

All claims expressed in this article are solely those of the authors and do not necessarily represent those of their affiliated organizations, or those of the publisher, the editors and the reviewers. Any product that may be evaluated in this article, or claim that may be made by its manufacturer, is not guaranteed or endorsed by the publisher.

## Footnotes

1
http://adni.loni.usc.edu

2
http://surfer.nmr.mgh.harvard.edu/fswiki

3
http://www.fil.ion.ucl.ac.uk/spm

4
http://restfmri.net

5
https://www.mathworks.com
