# Supplementary material for: Predicting MCI to AD Conversation Using Integrated sMRI and rs-fMRI: Machine Learning and Graph Theory Approach
Source: Front Aging Neurosci. 2021 Jul 30;13:688926. doi: 10.3389/fnagi.2021.688926 (PMC8375594; doi:10.3389/fnagi.2021.688926)
Supplement: Supplementary file 1 [file Data_Sheet_1.pdf]

Supplementary Materials

1. In Figure 1a, Figure1b, the top 30 features of the two groups (MCInc vs. MCIc, MCIc vs. AD) were selected by the RSFS, SS-LR, and mRMR in the cost = 8 – 44%. These classification results showed that the AUC and ACC obtained by the RSFS algorithm were significantly higher than the other algorithms

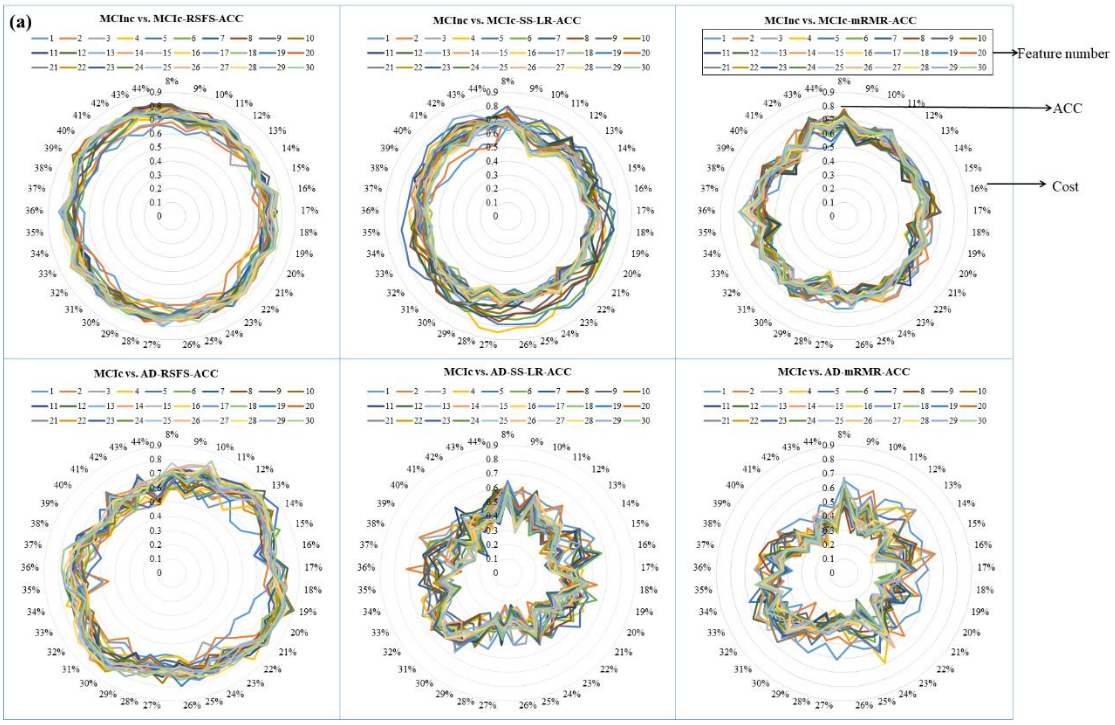

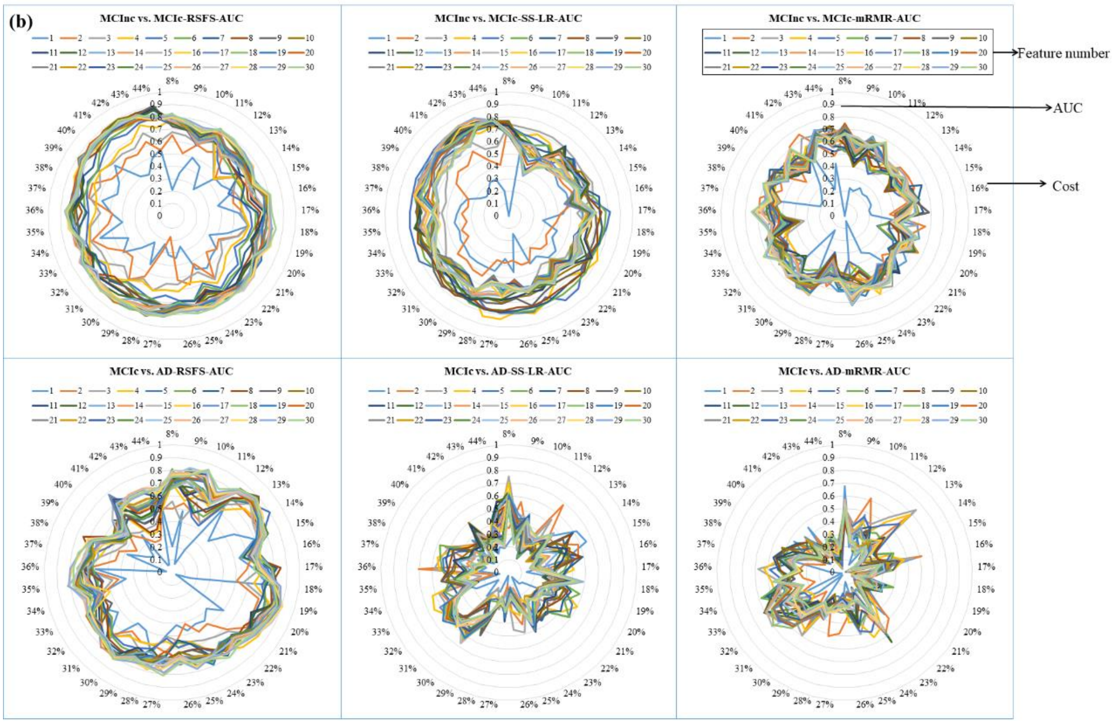

2. We compared the performance of multiple classifiers and verified the reliability of our results through upsampling. As shown in **Figure 2**, the oversampled data were trained and tested by four classifiers (Random Forest (Breiman, 2001), KNN (Yang et al., 2007), AdaBoost (Hastie et al., 2009), SVM).

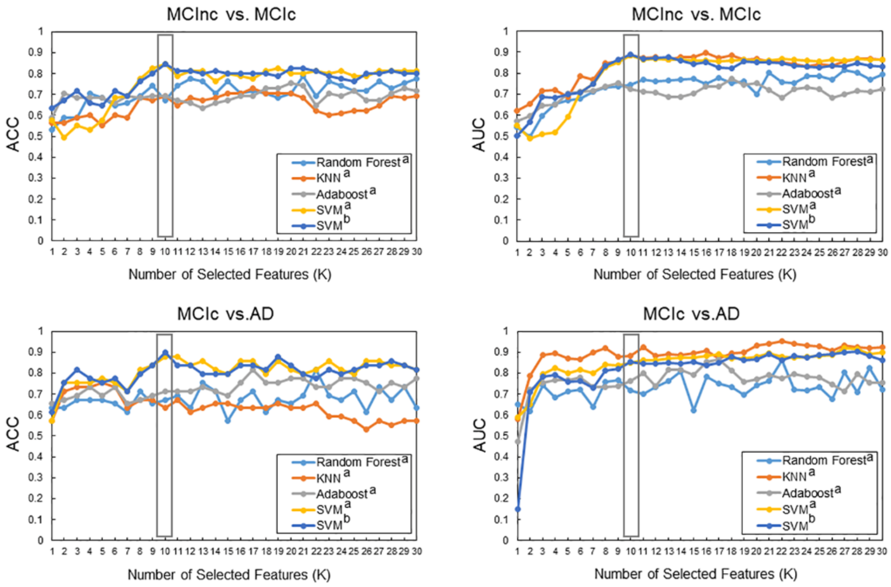

As illustrated in **Figure 3, Figure 4**, these results show that the result classification performance of the original nosampling data is between upsampling and downsampling when the number of features is 1-30.

Figure 3.

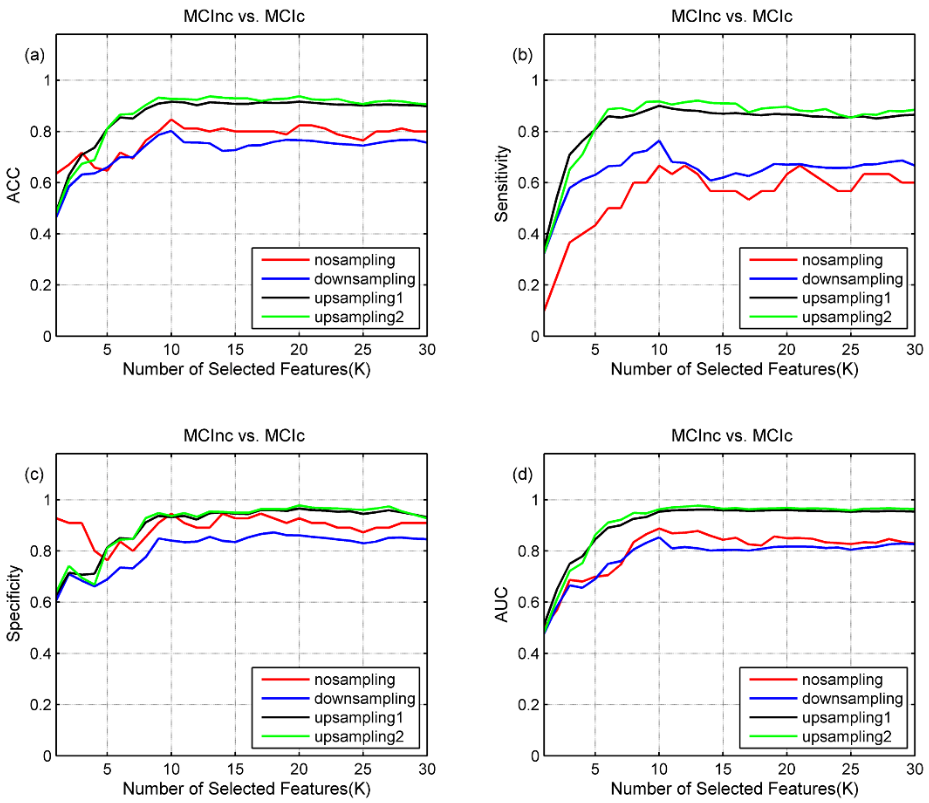
MCInc vs. MCIc
(a)
ACC
(b)
Sensitivity
(c)
Specificity
(d)
AUC
Number of Selected Features(K)
nosampling
downsampling
upsampling1
upsampling2

Figure 4.

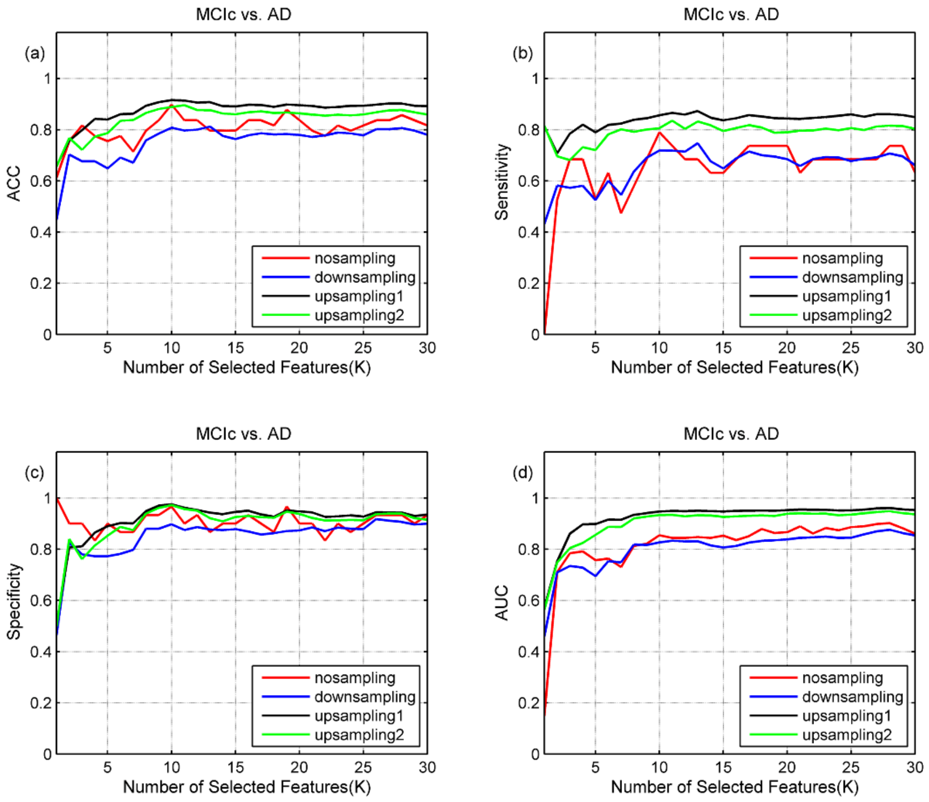
MCIc vs. AD
(a)
ACC
(b)
Sensitivity
(c)
Specificity
(d)
AUC
Number of Selected Features(K)
nosampling
downsampling
upsampling1
upsampling2

3. Table 3.1 | Selected feature distributions in the MCInc vs. MCIc group using the mRMR algorithm

Feature index	Modality	Frequency band	Networks attribution	Region	Frequency (%)
27	structural		thickness	SFG.L	100
208	SCN		ND	CUN.R	100
658	FCN	slow-4	NL	LING.R	100
1013	FCN	slow-5	ND	CAL.L	100
539	FCN	full band	BC	SMA.L	98.82
557	FCN	full band	BC	HIP.R	95.29
669	FCN	slow-4	NL	SPG.R	80.00
14	structural		thickness	IFGtri.R	78.82
826	FCN	slow-4	BC	CAU.R	77.65
76	structural		volume	MFG.R	42.35

SFG.L: left superior frontal gyrus; **CUN.R**: right cuneus cortex; **LING.R**: right lingual gyrus; **CAL.L**: left calcarine fissure and surrounding cortex; **SMA.L**: left supplementary motor area; **HIP.L**: left hippocampus; **SPG.L**: left superior parietal gyrus; **IFGtri.R**: right inferior frontal gyrus, triangular part; **CAU.R**: right caudate nucleus; **MFG.R**: right middle frontal gyrus

Table 3.2 | Selected feature distributions in the MCInc vs. MCIc group using the SS-LR algorithm

Feature index	Modality	Frequency band	Networks attribution	Region	Frequency (%)
1	structural		thickness	BSTS.L	100
27	structural		thickness	SFG.L	100
39	structural		thickness	ENT.L	100
69	structural		volume	BSTS.L	100
489	FCN	full band	ND	SPG.L	100
759	FCN	slow-4	ND	SPG.L	100
1029	FCN	slow-5	ND	SPG.L	90.59
3	structural		thickness	cMFG.L	80.00
760	FCN	slow-4	ND	SPG.R	80.00
669	FCN	slow-4	NL	SPG.L	56.47

BSTS.L: left banks superior temporal sulcus; **SFG.L**: left superior frontal gyrus; **ENT.L**: Entorhinal cortex; **SPG.L**: Superior parietal gyrus; **cMFG.L**: Caudal middle frontal gyrus; **SPG.R**: Superior parietal gyrus

Table 3.3 | Selected feature distributions in the MCIc vs. AD group using the mRMR algorithm

Feature index	Modality	Frequency band	Networks attribution	Region	Frequency (%)
287	SCN		NL	PHG.L	100
523	FCN	full band	BC	SFGdor.L	100
562	FCN	full band	BC	AMYG.R	100
581	FCN	full band	BC	IPL.L	97.96
934	FCN	slow-5	NL	IOG.R	93.88
413	FCN	full band	NL	PUT.L	89.80
821	FCN	slow-4	BC	ACG.L	85.71
408	FCN	full band	NL	PCUN.R	77.55
658	FCN	slow-4	NL	LING.R	32.65
353	FCN	full band	NL	IFGtri.L	24.49

HIP.L: left hippocampus; **SFGdor.L**: left **s**uperior frontal gyrus, dorsolateral; **AMYG.R**: right Amygdala; **IPL.L**: left inferior parietal, but supramarginal and angular gyri; **IOG.R**: right Inferior occipital gyrus; **PUT.L**: left lenticular nucleus, putamen; **ACG.L**: left anterior cingulate and paracingulate gyri; **PCUN.R**: right precuneus**;** **LING.R**: right lingual gyrus; **IFGtri.L**: left inferior frontal gyrus, triangular part;

Table 3.4 | Selected feature distributions in the MCIc vs. AD group using the SS-LR algorithm

Feature index	Modality	Frequency band	Networks attribution	Region	Frequency (%)
778	FCN	slow-4	ND	THA.R	100
1033	FCN	slow-5	ND	SMG.L	100
1066	FCN	slow-5	BC	ORBsup.R	100
1082	FCN	slow-5	BC	OLF.R	100
589	FCN	full band	BC	PCL.L	97.96
648	FCN	slow-4	NL	HIP.R	83.67
1001	FCN	slow-5	ND	ACG.L	79.59
440	FCN	full band	ND	ORBmid.R	75.51
1057	FCN	slow-5	ND	STG.L	67.35
523	FCN	full band	BC	SFGdor.L	61.23

THA.R: right thalamus; **SMG.L**: left supramarginal gyrus; **ORBsup.R**: right superior frontal gyrus, orbital part; **OLF.R**: right olfactory cortex; **PCL.L**: left paracentral lobule; **HIP.R**: right hippocampus; **ACG.L**: left anterior cingulate and paracingulate gyri; **ORBmid.R**: right middle frontal gyrus orbital part; **STG.L**: left superior temporal gyrus; **SFGdor.L**: left superior frontal gyrus, dorsolateral
